# Syndromic Surveillance—Review on Different Practices’ Performance and Added Value for Public Health

**DOI:** 10.3390/epidemiologia7010023

**Published:** 2026-02-03

**Authors:** Zhivka Getsova, Vanya Rangelova

**Affiliations:** 1 Epidemiology Department, National Center of Infectious and Parasitic Diseases, 1504 Sofia, Bulgaria; 2 Center of Competence ImmunoPathogen, 1504 Sofia, Bulgaria; 3 Department of Epidemiology and Disaster Medicine, Faculty of Public Health, Medical University of Plovdiv, 4000 Plovdiv, Bulgaria; vanya.rangelova@mu-plovdiv.bg

**Keywords:** syndromic surveillance, infectious disease, early warning, epidemic preparedness, data integration

## Abstract

Timely identification of infectious disease threats is essential for public health readiness. Conventional indicator-based surveillance systems, while dependable for tracking established pathogens, frequently lack the agility and sensitivity to detect new infections promptly. Syndromic surveillance, which examines pre-diagnostic and non-specific health indicators from many data sources in near real time, serves as a significant complementary method that improves early warning and situational awareness. This narrative study analysed selected experiences with syndromic surveillance, utilising peer-reviewed literature and institutional records. Four primary data streams were examined: emergency department and hospital records, pharmacy and over the counter (OTC) sales, participative citizen-generated data, and hybrid multi-source systems. Syndromic indicators were reported to identify outbreaks two to fourteen days before standard laboratory reporting across many trials. Data from the emergency department exhibited the highest sensitivity and specificity (47.34% and 91.95%, respectively), whereas pharmacy and participative data offered early indicators at the community level. Integrated systems like ESSENCE (Johns Hopkins University Applied Physics Laboratory, Laurel, MD, USA) and SurSaUD^®^ (Saint-Maurice cedex, Paris, France) attained enhanced accuracy yet necessitated significant data integration and governance capabilities. Syndromic surveillance enhances epidemic preparation by detecting atypical health-seeking behaviours and variations from baseline patterns prior to formal diagnosis. Nonetheless, its efficacy is contingent upon data quality, interoperability, and contextual adaptation. Countries like Bulgaria could improve national early-warning capabilities and overall health security through the gradual adoption of pilot projects and integration with existing surveillance networks.

## 1. Introduction

Epidemiological surveillance is defined as “the ongoing, systematic collection, analysis, and interpretation of health data essential to the planning, implementation, and evaluation of public health practice, closely integrated with the timely dissemination of [this information] to those who need to know.” [1]. Transformed into epidemiological information, it is the basis for planning and conducting control programmes and evaluating their effectiveness.

Since the revision of the International Health Regulations in 2005, the role of surveillance as a major component of public health has increased [2]. For this purpose, the collected information is analysed, interpreted, and the results are widely disseminated. The frequency and prevalence of infectious diseases, as well as the main determinants of diseases, are tracked. In this way, a type of safety net is created, incidents are identified and trends in the development of infectious diseases are tracked.

The data acquired from surveillance systems serves three interconnected purposes. The primary purpose of this data is to quickly identify and respond to epidemics or emerging health concerns that require immediate containment measures. Secondly, for the systematic evaluation and monitoring of essential health indicators—such as illness prevalence, immunisation rates, and antibiotic resistance—which provide trend analysis, benchmarking, and adjustments to preventive measures. Moreover, effective surveillance facilitates evidence-based decision-making and permits the assessment of the impact and effectiveness of public health interventions.

Public health needs, resource restrictions, and governance arrangements determine surveillance system design across situations and countries. Given current surveillance methods, traditional systems are not sensitive enough to detect emerging diseases and clusters [3]. Despite prompt notification, the connection to a specific disease may not always be evident, potentially leading to a delayed diagnosis. Second, the aetiological link between initial clinical presentation and a notifiable disease may be unclear, delaying case confirmation even when reporting is timely. Third, while decentralising health services is meant to improve efficiency, it might fragment information flow and lower data quality, making surveillance coordination harder [4]. Modern guidance recommends the digitalisation, interoperability, and real-time integration of current systems to speed up data collection and turnaround [5].

Based on the models for collecting information, the surveillance of diseases can be divided into indicator-based surveillance (IBS), event-based surveillance (EBS), and syndromic surveillance.

The most utilised system for disease detection and reporting is disease-specific surveillance, or IBS, which involves routine notification of diseases and health events through notifiable disease systems. Many countries maintain a predefined list of diseases or conditions of interest, which use standardised case definitions to ensure consistency and comparability.

In contrast, EBS aggregates ad hoc signals from laboratories, media reports, digital platforms, and other non-traditional sources, thereby complementing IBS by detecting unusual occurrences that fall outside formal reporting pathways. Integrating IBS with event-based components can markedly improve sensitivity, specificity, and overall responsiveness [6]. In a period that is marked by rapid pathogen introduction and increased global interconnection, such advancements are essential for satisfying the core-capacity requirements of the International Health Regulations (2005) [2] and for protecting the health of the public.

The third type of surveillance, which can be utilised—syndromic surveillance—is based on non-specific health indicators collected for purposes other than surveillance and, where possible, are automatically generated for allowing early detection of human or veterinary public health threats [7]. The alterations in the epidemiology of infectious diseases enable the creation of a syndromic surveillance system to meet the new demands. By transcending established norms, the syndrome-based approach enhances the sensitivity of case detection relative to alternative methods.

### Identification of Contemporary Needs

Early diagnosis of novel or emerging infectious diseases is crucial to public health emergency response. Global warming and armed conflict have changed the climate and social backdrop, requiring public health to adapt to biological agent uncertainty and even deliberate usage [8]. Climate change is rapidly changing the ecology of several viruses and their vectors [9,10,11], expanding the potential distribution of West Nile virus, dengue, chikungunya, and vibrio infections into temperate regions. West Nile virus may increase three- to five-fold in Western Europe under moderate-to-high emission scenarios by mid-century, endangering millions of immunologically naïve individuals, as seen in the UK in 2025 [12]. In this environment, modern surveillance systems must detect diseases in unusual settings and seasons. Epidemic control requires flexible, real-time detection. When community immunity is low and healthcare resources are scarce, even a few-day delay in diagnosing unusual clinical clusters increases transmission risk. Thus, modifying monitoring systems for rapid response to vector-borne arboviruses in changing climates or novel respiratory pathogens has become a global health security priority.

Recently, the need to develop rapid response capabilities has been starkly illustrated by two high-impact events: the COVID-19 pandemic [10,13,14] and the unprecedented 2022 outbreak of Mpox registered on the territory of Europe and North America. Both diseases meet the criteria for new or re-emerging infections, yet each revealed different facets of the same vulnerability—how quickly an undetected pathogen can propagate when early-warning signals are missed.

SARS-CoV-2, the cause of COVID-19, was unknown to medicine until 31 December 2019, when physicians in Wuhan, China, reported a cluster of severe, unexplained pneumonia [15]. The virus was genetically described and named on 11 February 2020, but sustained community transmission was already global, highlighting the short window for containment once gradual spread began [16,17,18,19]. Metrics including mortality, lethality, and excess mortality vary due to country-level monitoring capacities and methods [17]. Mpox outbreaks in 2022 occurred in Europe and North America, where none had occurred before. The WHO declared a Public Health Emergency of International Concern after over 100 nations reported infections in three months [20].

These two cases demonstrate the limitations of IBS. Conventional methods identify diseases that are well-defined and predictable within particular clinical or geographic criteria; syndromic manifestations that deviate may go undiscovered until an epidemic is confirmed. In early-alert systems, limiting reporting requirements reduces sensitivity to healthcare use anomalies while delaying diagnosis [21,22].

Public health practice is moving towards multi-source, real-time surveillance systems that integrate electronic health records, over-the-counter pharmaceutical sales, social media discussions, and open-source data to close this detection gap. Integrating heterogeneous data, including pre-diagnostic data on symptoms before laboratory confirmation, increases the likelihood of detecting weak, atypical signals like abrupt increases in non-specific respiratory complaints or dermatological consultations before laboratory confirmation. COVID-19 and Mpox provide significant empirical support for broad-spectrum early-warning systems that use thresholds and activate for validation when critical values are exceeded. These systems should recognise unusual health-seeking behaviours across channels to reduce signal-to-response time and disease transmission [5].

Given the diversity of syndromic surveillance approaches, this work compares the performance of commonly applied practices to suggest an understanding of each model’s added value.

## 2. Materials and Methods

This article is a selective, illustrative narrative review that comparatively examines syndromic surveillance methodologies and their applications in public health. The aim was not to provide an exhaustive or fully systematic synthesis of all available studies but rather to integrate representative peer-reviewed evidence and practical experiences to explore the reported benefits, limitations, and strategic roles of syndromic surveillance systems in contemporary public health practice.

The review was guided by the hypothesis that syndromic surveillance systems typically demonstrate high sensitivity but variable baseline specificity and sought to explore whether syndromic signals primarily complement traditional surveillance activities or introduce additional operational burden through false-positive alerts and increased informational noise.

### 2.1. Literature Identification and Selection

Relevant peer-reviewed literature, technical reports, and institutional publications were identified through targeted electronic searches of PubMed, Scopus, and Web of Science. Searches were conducted using combinations of terms related to syndromic surveillance, participatory surveillance, emergency and primary care data for public health, automated surveillance systems, ICD (International Classification of Diseases) code utility for early detection, pre-diagnostic chief complaint data, early warning indicators, mass gatherings surveillance, and non-prescription medication sales. The search was limited to publications from 2000 to 2025, which reflects the period during which syndromic surveillance has gained considerable global prominence, undergone substantial methodological development, and been implemented worldwide.

Study selection followed a purposive, topic-driven approach typical of narrative reviews. Publications were included if they:

- described syndromic or participatory surveillance systems or methods;
- reported empirical findings on performance characteristics (e.g., sensitivity, specificity, timeliness) or operational utility;
- provided methodological insights, evaluations, or applied case studies relevant to public health surveillance.

Publications were excluded if they:

- did not involve human or population-level surveillance data;
- focused exclusively on laboratory-confirmed surveillance without a syndromic component;
- lacked sufficient methodological or contextual detail to inform comparative discussion.

Formal quality scoring or risk-of-bias assessment was not performed, as the purpose of the review was interpretive rather than evaluative.

### 2.2. Data Synthesis and Interpretation

Evidence was synthesised narratively, with studies grouped thematically according to data source, surveillance setting, analytical approach, and public health application. Conflicting findings across studies were discussed qualitatively, with attention to contextual factors such as surveillance purpose, data source, outbreak type, and health system setting.

Where numerical performance measures (e.g., sensitivity, specificity, lead time) are summarised across studies, these values represent descriptive, illustrative aggregates drawn from heterogeneous sources. They are descriptive estimates, and one should not interpret them as meta-analytic summaries because they were not pooled or weighted by study size or quality. Their purpose is to provide an indicative range of reported performance, rather than precise quantitative benchmarks.

This study utilised only publicly accessible secondary data; therefore, ethical approval was not required.

## 3. Results and Discussion

While much of the reviewed literature emphasises the supposed added value of syndromic surveillance for early signal detection, the evidence base is mixed, and reported benefits are highly contingent on system design, governance, and response capacity.

### 3.1. International Experience in Syndromic Surveillance Implementation

#### Syndromic Surveillance Based on Emergency Department and Hospital Data

Addressing the deficiencies in conventional notifiable-disease systems requires a clearly delineated multi-channel framework that simultaneously employs clinical, laboratory, administrative, and unconventional data sources [5]. The conceptual basis for this technique was established shortly after the 11 September 2001, attacks, when U.S. public health organisations investigated the potential of near-real-time logs from emergency department admissions as an early-warning indicator for significant bioterrorism incidents. The discussions—ultimately formalised by systems like BioSense and ESSENCE—revealed that passive, clinician-initiated reporting is inherently less informative than the ongoing electronic collection and automated analysis of comprehensive Emergency Department (ED) registries [23,24]. Selective submission may exclude clinically mild or unusual symptoms, thereby creating systematic gaps and obscuring the true onset of an outbreak.

The latency inherent in conventional case-notification processes is equally concerning; individual records must initially meet case-definition criteria, followed by validation, transmission, and manual curation before aggregate analysis can commence. This administrative delay, frequently quantified in days, diminishes the temporal resolution required for swift intervention and ultimately compromises confidence in the resultant situational assessment [25].

In 2002, the experience of multiple healthcare facilities in the USA validated a model that categorised complaints or illnesses based on ICD-9 codes, so confirming the benefits of syndromic surveillance. Espino’s study indicated that the screening sensitivity was 44% and the specificity was 97%, with the analysis of reported complaints (diagnosed before confirmed diagnosis) demonstrating the highest significance relative to the clinical diagnosis [26]. The methods of processing pre-diagnostic data based on symptoms, without pre-diagnostic coding, exhibited contrasting results. At a specificity threshold of 93%, the sensitivity of pre-diagnostic data compared to the confirmed diagnosis was 2 to 3% lower [27]. Nonetheless, the integration between the two models is characterised by enhanced accuracy [27]. Data availability for specific diseases associated with highly specific disease manifestations shapes findings related to the efficiency of syndromic surveillance. Highly generic symptoms, such as those reported for acute respiratory infections, are expected to score high on sensitivity but low on the specificity scale [28], while specific symptoms and exposure history relevant to some syndromes, such as gastroenteritis, were pointed to as leading to immediate diagnostic implications [29]. An explanation for the inaccuracies associated with symptom assessment is provided by the findings of Roberts’ research team, which indicate that the same disease may present with varying symptoms among different patients. Expanding the definitions, as proposed by the implementation of operational ICDs, may address the identified issue [30]. 

### 3.2. Syndromic Surveillance—Basic Indicators, Approaches and Utility

Contemporary medical practice aspires to accuracy, indicating one or multiple working diagnoses during a single visit. Most reports on syndromic surveillance provide data on ICD-9 codes, working diagnoses, free-text symptoms, sex, patient residence, and visit locations; all of these reports also include an ICD-9 clinical diagnosis, as well as age and visit time [31,32]. The reviewed literature evaluates the data analysis in relation to the potential for early detection of national security threats stemming from the intentional deployment of biological agents.

In the 2002 Winter Olympic Games in Utah, a comparable system was implemented, wherein the patient numbers from prior periods were analysed against those registered for the corresponding current period. Values surpassing the 95% confidence interval were deemed indicative of concern about mitigating false alarms [25,33]. Practical experience confirms thresholds may be modified based on a prior risk assessment [34], resulting in tailored risk-based surveillance models allowing for resource allocation according to emergency needs [35]. Various syndromic surveillance systems employ distinct methodologies for syndrome partitioning based on their research [25,36,37,38]. The age of the patients is a significant variable to consider. Changes in disease demographics may indicate the emergence of a new infection, as evidenced by the study conducted in Syria during the COVID-19 pandemic [39].

### 3.3. Pharmacy and Over-the-Counter (OTC) Sales Data

Trends in pharmaceutical sales provide a significant amount of preclinical data. A rise in over-the-counter drug sales—such as antipyretics, cough suppressants, or gastrointestinal treatments—can act as an early indicator of increasing disease prevalence in the community [25]. Mandl’s surveillance model posits that these data sources preceded emergency department utilisation, illustrating the inherent latency between symptom onset and individuals’ decisions to pursue medical care [34]. Pharmacy-based sales metrics reflect the behavioural changes that take place when individuals initially encounter symptoms and pursue over the counter (OTC) relief [34]. Numerous studies have identified these indicators as easy to collect, epidemiologically significant, and timely enough for public health decision-making [40,41,42,43,44,45,46].

The literature accessed indicates that deviations in pharmaceutical demand frequently arise prior to similar indicators appearing in standard clinical data. Sales increases in antidiarrheal or rehydration products have been observed approximately two weeks prior to clinically confirmed diarrheal outbreaks [45]. In contrast, unusual acquisitions of antipyretics, cough suppressants, or other respiratory treatments generally result in emergency department identifications occurring approximately 2.8 days later (range: 2–7 days) [46]. Das et al. [47] indicate that, despite the advantageous lead time, the overall sensitivity of pharmacy-based surveillance is inferior to that of emergency department visit counts, primarily due to variations in performance associated with disease aetiology [47]. OTC sales may inadequately reflect pathogens that produce mild or ambiguous early symptoms, while they are more effective for conditions that necessitate prompt self-treatment, such as influenza-like illness.

The predictive value of pharmaceutical sales data is context-specific, influenced by cultural norms, socioeconomic factors, healthcare-seeking behaviour, and the structure of national pharmacy markets. In contexts characterised by prevalent self-medication and well-established digital reporting systems in pharmacies, sales data can serve as an early and cost-effective indicator. In contrast, in nations with robust physician-gatekeeper systems or restricted over the counter access, the signal may be weak or delayed. A Japanese study indicated minimal additional benefit from monitoring pharmacy transactions for influenza spread, likely due to local consultation patterns and dispensing regulations [48].

### 3.4. Participatory and Citizen-Generated Data

Alongside conventional clinical data acquired from primary care providers, preclinical data sources (patient- and OTC sales-generated information) give significant insights for early detection. One such source is citizen-generated information, wherein citizens report and evaluate the severity of newly encountered symptoms linked to infectious diseases. Despite the limited adoption of this data in public health practice, research during seasonal influenza outbreaks and subsequent COVID-19 waves has revealed a strong association between self-reported symptoms and laboratory-confirmed illnesses [49,50,51].

A significant benefit of participatory surveillance systems is their flexibility and adaptability. These systems depend on voluntary contributions from the general populace, allowing for facile configuration to monitor a wide range of ailments. This method has demonstrated efficacy not just during respiratory virus epidemics but also in situations of increased threat from infections like the Zika virus and *Salmonella* species [50].

### 3.5. Other Innovative and Hybrid Models

Syndromic surveillance is particularly effective in identifying emerging or previously undiagnosed diseases prior to formal clinical diagnosis. Experts recommend aligning monitoring algorithms with established patterns of disease seasonality and cyclicity to ensure that these systems deliver meaningful and timely alerts. By considering anticipated variations within standard epidemiological parameters, the system can concentrate on detecting genuinely anomalous events, thereby minimising false-positive signals [34].

**Figure 1:**
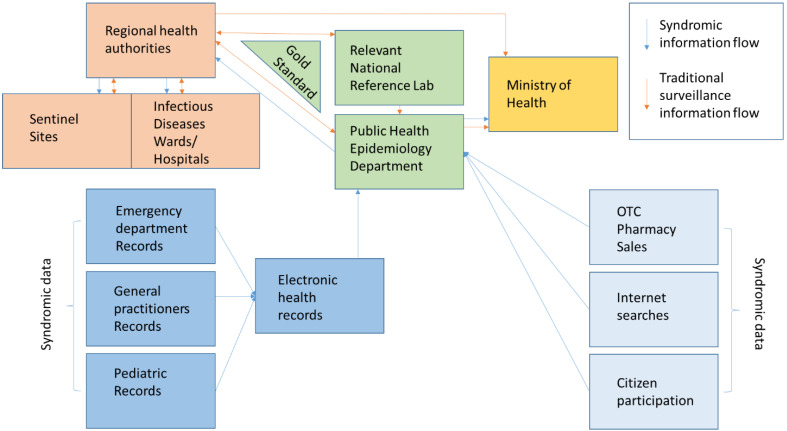
Suggested model of hybrid/multi-source system information exchange [23,24,25,26,27,28,29,30,31,32,33,34].

Empirical studies of the late 20th and early 21st centuries set out to clarify the utility of “unorthodox” pre-diagnostic data for early disease detection by expanding their scope to include pharmacy sales and Internet searches. The methods used are simple and are limited to determining a critical threshold for each indicator. Deviations from it are subject to in-depth analysis. Wagner’s study of different surveillance methods uses an influenza A epidemic as a subject, and it aims to measure the timeliness of different indicators to detect a peak in morbidity. The gold standard is two consecutive weeks in which more than 1% of the samples were positive for the presence of the pathogen of interest. His measured sensitivity ranged from 32% (for paediatric referrals with flu-like symptoms) to 81% (for emergency room visits). Specificity ranges from 63% (sentinel reports) to 75% (emergency department visits). The results showed that emergency examination data were not only the most accurate but also ahead of the gold standard by 4 weeks, while paediatric care seeking was delayed by 5 weeks compared to the gold standard [33]. This finding regarding the early timeliness of the emergency medical care demand indicator is supported by other studies that conclude that data is applicable to increasing hospital care capacity prior to entering a peak wave [52,53]. The main advantage of emergency and outpatient registries is the prior availability of data on prevalent syndromes [34]. Figure 1 summarises platform integration and communication channel establishment according to the literature sources [23,24,25,26,27,28,29,30,31,32,33,34].

Table 1 summarises the comparative advantages and limitations of the main data sources used in syndromic surveillance. Mean values from the different research processes point to an average sensitivity and specificity of 52.64% and 87.67% of different syndromic surveillance approaches. Emergency department (ED) data solely had an average of 47.34% sensitivity and 91.95% specificity, according to the five different sources reporting measurements for these indicators. Average measures of timeliness from the analysed literature show a lead time of 5.75 days.

Across studies, syndromic surveillance consistently offers earlier outbreak detection (2–14 days ahead of laboratory confirmation). Emergency department-based systems exhibit advantages in terms of timeliness as well as the highest sensitivity, while pharmacy sales and participatory data enhance early community-level awareness. Multi-source systems achieve the best balance between sensitivity and specificity but demand significant infrastructure and data-governance capacity. The efficacy of syndromic surveillance is significantly contingent upon data quality, integration, and contextual interpretation. Systems devoid of defined standards, compatible platforms, or consistent data inputs are susceptible to generating false alarms or overlooking critical signals. The equilibrium between sensitivity and specificity continues to be a fundamental scientific difficulty. Although expansive syndromic criteria can enhance early signal detection, they may also produce false positives that strain public health resources. Correspondingly, ethical and legal problems about personal data utilisation and privacy safeguarding must be tackled via transparent governance and comprehensive data-protection frameworks. Importantly, the ability of syndromic surveillance systems to detect early signals does not inherently translate into effective public health response, as response capacity is shaped by organisational readiness, governance, and resource availability beyond the surveillance system itself.

Incorporating artificial intelligence (AI) and machine-learning algorithms into syndromic surveillance can improve forecasting capabilities and anomaly identification. These devices can autonomously detect variations from baseline patterns and minimise response time during outbreaks. Syndromic surveillance possesses significant potential to enhance national and regional health security frameworks, contingent upon its integration into a well-coordinated and EBS ecosystem [55,56,57].

### 3.6. Limitations

This narrative review has several limitations. The literature selection was selective and illustrative rather than comprehensive, and formal risk-of-bias assessment was not undertaken. Quantitative summaries are intended to convey indicative ranges reported in the literature and do not represent pooled or weighted estimates. Substantial heterogeneity in surveillance settings and methodologies may limit the generalisability of specific conclusions.

Future research should focus on assessing syndromic monitoring via pilot deployments that evaluate accuracy, timeliness, and public health impact, while investigating integration with AI-driven predictive analytics.

## 4. Conclusions

Different monitoring technologies are affected by biological and sociocultural factors. Social norms and infection prevalence can affect syndromic surveillance outcomes spatially. The accuracy of the initial risk assessment and the criteria set must be updated based on national or regional seasonality and cyclicality to make the strategy effective.

The implementation of syndromic surveillance relies on the integration of various electronic systems and collaboration among the institutions that provide data to the platforms. The aggregation of data on syndromes should reflect prior risk analysis to improve pandemic readiness.

## Figures and Tables

**Table 1:** Comparative advantages and limitations of syndromic surveillance data sources.

Data Source	Example System/Study	Lead Time (vs. Lab Confirmation)	Sensitivity/Specificity	Advantages	Limitations
Emergency Department Records	Espino et al. [26]	4–7 days earlier	Sensitivity 44%, Specificity 97%	Timely, structured, standardised ICD coding	Requires integration across facilities; may miss mild cases
Emergency Department Records	Morbey R. [28]	Up to 7 days earlier	100% sensitivity for seasonal influenza and 0% for seasonal adenovirus.	Early; adaptable	highly dependent on system organisation
Emergency Department Records	Reis, B. Y., & Mandl, K. D. [27]	1-day detection approach	Sensitivity 26–47% Specificity 93%	Early information available in real-time, with the possibility to incorporate different data sources (symptom-based/diagnostic); temporal smoothing filters might reduce noise.	Chief complaints: lower accuracy
Emergency Department Records	Guasticchi G, et al. [29]	-	Variable, disease-specific: Sensitivity 22.2–90.2	Information routinely collected, automated	Results are highly dependent on syndrome definition.
Emergency Department Records	Rosenkötter N, et al. [54]	Up to 8 days earlier	Variable depending on country and source: Sensitivity 0–100%, Specificity 57.1–88.9%	Strong validity and timeliness of data	Results differ across countries due to differences in catchment population. In some cases, the identification of events is later than the references.
Pharmacy/OTC Sales	Das et al., 2005 [47]; Hogan et al., 2003 [44]	2–14 days earlier	Variable, disease-specific	Early community signal; inexpensive; easy to automate	False positives; context-dependent on self-medication patterns
Participatory (Citizen Reports)	Chunara et al., 2015 [49]; Mahmud et al., 2021 [51]	3–5 days earlier	High correlation with lab-confirmed data	Engages public; rapid; adaptable	Participation bias; data validation required
Hybrid/Multi-source Systems	Foldy et al., 2004 [24]	2–10 days earlier	Improved combined sensitivity	Comprehensive, scalable and high analytic power	Requires strong IT infrastructure and privacy safeguards

ICD: International Classification of Diseases; OTC: over the counter; IT: Information Technology.

## Data Availability

No new data were created or analysed in this study.
